# Successful rechallenge with azacytidine and venetoclax after sustained treatment-free remission in a relapsed acute myeloid leukemia patient: a case report

**DOI:** 10.1007/s00277-024-05922-6

**Published:** 2024-08-03

**Authors:** E. Tamellini, C. Simio, A. Bernardelli, I. Ferrarini, A. Vatteroni, A. Moioli, V. Macaluso, E. Marchetti, I. Tanasi

**Affiliations:** 1 Department of Engineering for Innovation Medicine, Section of Hematology, University of Verona, Verona, Italy; 2 Hematology and Bone Marrow Transplant Unit, Azienda Ospedaliera Universitaria Integrata di Verona, Verona, Italy

**Keywords:** Acute myeloid leukemia, Venetoclax, Azacytidine, Retreatment, Rechallenge, Treatment-free remission

## Abstract

Combined therapy with venetoclax and hypomethylating agents has significantly improved the outcome of unfit patients ineligible for intensive chemotherapy. A recently published exploratory analysis of the VIALE-A trial reported that up to 51% of patients achieving remission survived more than 2 years. These data along with those from reallife settings, lead to questioning how long it is appropriate to continue treatment in long-term survivors. Accordingly, recent retrospective studies suggested the feasibility of suspending therapy in selected patients while maintaining prolonged responses. Also, these studies showed that retreatment may induce a second remission in almost a third of patients. We report the case of a patient who received salvage therapy with venetoclax and azacytidine, that was discontinued few cycles after blasts clearance because of severe hematological toxicity. Despite suspension, he maintained a sustained response lasting almost one year and was successfully retreated with the same combination when a second relapse occurred.

Dear editor,

Venetoclax (VEN) in combination with hypomethylating agents (HMA) revolutionized acute myeloid leukemia (AML) treatment for elderly or unfit patients ineligible for intensive chemotherapy [1, 2]. Combined therapy with VEN and HMA is usually continued until disease progression, but recent retrospective studies highlighted the possibility of discontinuing treatment after achieving complete remission while preserving long-term response [3 ,4, 5].

Besides, anecdotal cases suggest the possibility of retreating prior responsive patients with the same VEN-based therapy at relapse [6, 7].

A retrospective study of a limited number of patients initially treated with VEN-HMA in first or subsequent lines of therapy, showed a response rate at retreatment of 33% (5 out of 11 patients, 3 CR and 2 CRi) [8].

We report the case of a patient with relapsed AML, who received salvage therapy with VEN-HMA and remained in CR after treatment discontinuation. When a second relapse occurred, the patient was successfully retreated with VEN-HMA, achieving a second CR.

In May 2018, a 66-year-old male was diagnosed with de novo AML, with NPM1 and FLT3-ITD mutations. The karyotype was normal. He underwent anthracycline-based intensive chemotherapy at our Center. By October 2018, he completed first-line therapy, achieving complete remission with negative NPM1 minimal residual disease (MRD). The patient refused further consolidation with allogeneic stem cell transplantation (allo-SCT) for personal reasons and started follow-up.

In October 2021, he experienced a late first disease relapse. Cytogenetic analysis revealed the acquisition of trisomy 8, whereas mutational analysis identified an IDH2 mutation and the disappearance of FLT3-ITD and NPM1 mutations. Second-line therapy with VEN-AZA (Azacytidine) was offered, as the patient categorically refused intensive chemotherapy. He started treatment in November 2021, but it was discontinued early in January 2022 after three cycles due to persistent severe cytopenia (Grade 4 neutropenia and Grade 4 thrombocytopenia). In April 2022, the CBC (Complete Blood Count) documented the complete hematological recovery, and the BM study confirmed the achievement of complete remission.

In April a second disease relapse occurred with same cytogenetic and molecular characteristics of the previous one.

In the absence of therapeutic alternatives, it was decided to resume therapy with VEN-AZA. A CR with partial hematological recovery was documented at the end of the first cycle. Nowadays, he remains under treatment and has completed 13 cycles of therapy, maintaining CR. The CBC is within normal ranges. Unfortunately, MRD assessment was not feasible as the patient had neither molecular nor cytofluorimetric markers.

Figure 1 summarizes patient’s history from diagnosis to VEN-HMA rechallenge.

**Fig. 1:**
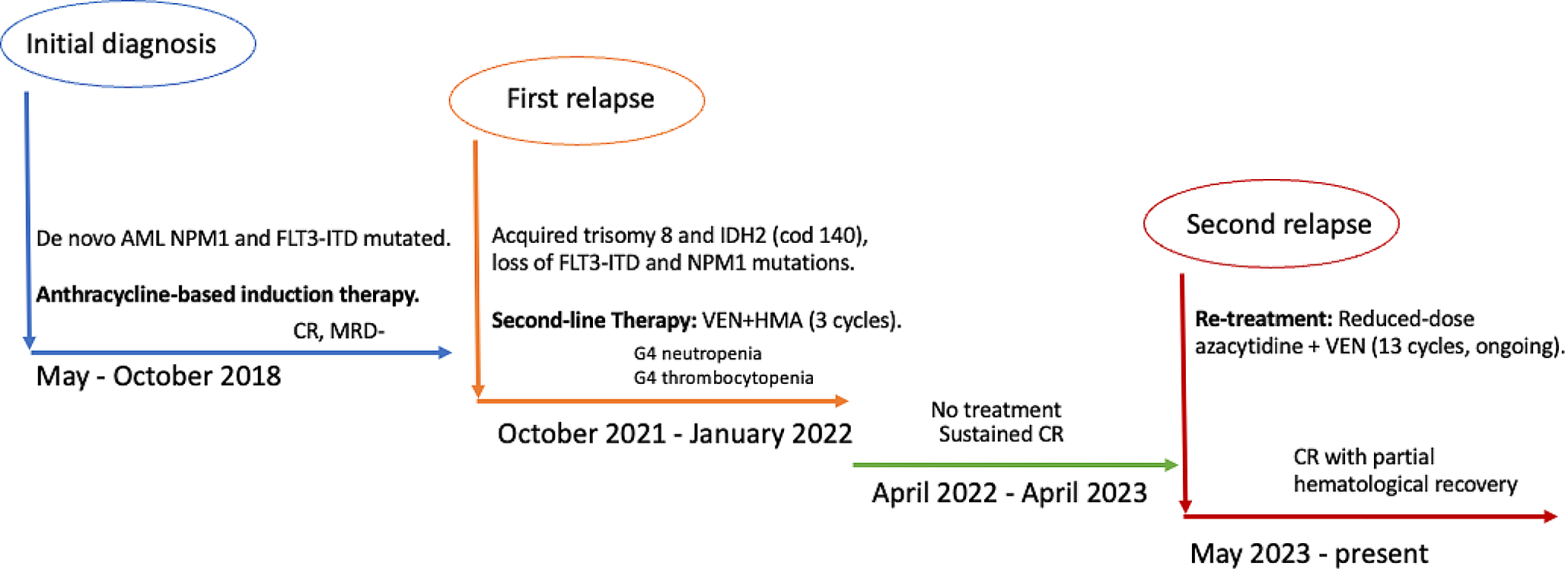
Summary of patient’s clinical history

An emerging question about VEN-HMA combination is whether treatment-responsive patients can discontinue therapy without compromising the duration of response and overall outcome. Retrospective analyses suggest that specific molecular features (i.e., NPM1, IDH1/2 mutated) and MRD negativity could be used as predicting factors for prolonged treatment-free remission, supporting the potential for disease eradication in select individuals. [3, 8]

Our case suggests that treatment discontinuation is feasible even in relapsed patients with reduced tolerability and is not necessarily associated with rapid loss of response. Furthermore, treatment rechallenge could be a viable option for subsequent relapse. Prospective trials are warranted to confirm these findings.

## Data Availability

No datasets were generated or analysed during the current study.
